# Cannabidiol and SARS-CoV-2 Infection

**DOI:** 10.3389/fimmu.2022.870787

**Published:** 2022-03-24

**Authors:** Alexandre Vallée

**Affiliations:** Department of Epidemiology-Data-Biostatistics, Delegation of Clinical Research and Innovation (DRCI), Foch Hospital, Suresnes, France

**Keywords:** COVID-19, Wnt/β-catenin pathway, PPARγ, ACE2, cannabidiol, SARS-CoV-2

## Abstract

Cannabidiol (CBD) can prevent the inflammatory response of SARS-CoV-2 spike protein in Caco-2-cells. This action is coupled with the inhibition of IL-1beta, IL-6, IL-18, and TNF-alpha, responsible for the inflammatory process during SARS-CoV-2 infection. CBD can act on the different proteins encoded by SARS-CoV-2 and as an antiviral agent to prevent the viral infection. Furthermore, recent studies have shown the possible action of CBD as an antagonist of cytokine release syndromes. In the SARS-CoV-2 pathophysiology, the angiotensin-converting enzyme 2 (ACE2) seems to be the key cell receptor for SARS-CoV-2 infection. The WNT/β-catenin pathway and PPARγ interact in an opposite manner in many diseases, including SARS-CoV-2 infection. CBD exerts its activity through the interaction with PPARγ in SARS-CoV-2 infection. Thus, we can hypothesize that CBD may counteract the inflammatory process of SARS-CoV-2 by its interactions with both ACE2 and the interplay between the WNT/β-catenin pathway and PPARγ. Vaccines are the only way to prevent COVID-19, but it appears important to find therapeutic complements to treat patients already affected by SARS-CoV-2 infection. The possible role of CBD should be investigated by clinical trials to show its effectiveness.

## Introduction

Several studies have been investigated to immunize or cure the COVID-19 disease. However, as the severe acute respiratory syndrome coronavirus-2 (SARS-CoV-2) evolved, new mutants can appear to infect and recombine their different hosts (1). While many of applied therapies are promising, they may induce some negative side effects (2, 3). Therefore, it is imperative to investigate new therapeutic strategies with effective treatment showing no or less side effects. In complement to vaccines, the only effective way in the prevention of COVID-19, natural agents may participate in SARS-CoV-2 (4, 5). One possible strategy is the use of cannabidiol (CBD) which exhibits anti-inflammatory and immune-suppressive effects in preclinical models of COVID-19. Nevertheless, very few studies have synthesized the different pathways which could explain the possible effects of CBD in SARS-CoV-2 infection. Thus, this review focuses on the different actions of CBD in SARS-CoV-2 infection and then the possible effects of CBD by interacting with both the WNT/β-catenin pathway and PPARγ expression in this viral disease.

## Cannabidiol

CBD, a member of the cannabinoid class produced by *Cannabis sativa*, presents many actions in diseases, as antiviral inflammatory responses (6). However, the biologic actions of CBD remain unclear (7). Different solutions of CBD have been approved as drug by the FDA in the USA, such as therapy in epilepsy (8). Few reports have shown the interest of CBD in SARS-CoV-2 infection while its beneficial effects have been observed in viral diseases, including hepatitis C (9, 10).

## CBD and the Proteins Encoded by SARS-CoV-2

The SARS-CoV-2 genome, which encodes for several proteins, needs to replicate itself to infect humans (11, 12). These proteins were, for example, SARS-CoV-2Mpro, glycoprotein (S), notorious spike (S) protein (recognizing ACE2 in the first step of infection), chymotrypsin-like main protease, papain-like protease, RNA polymerase (synthesizing viral RNA), and the RNA-cleaving endoribonuclease (responsible for SARS-CoV-2 progression) (12).

A recent study presented an interesting finding in natural products, as cannabidiol (CBD) for the treatment of the COVID-19 disease (13). In their study, they found that CBD and its metabolite 7-OH-CBD can block SARS-CoV-2 replication. CBD acts after viral entry by reversing the transcription of host genes and their expression. CBD can increase the IRE1-alpha RNase endoplasmic reticulum stress response and the interferon pathways (13). The primary target for entry into host cells has been identified to be the multifunctional protein angiotensin-converting enzyme-related carboxypeptidase (ACE2) discovered simultaneously by Donoghue et al. and Tipnis et al. (14, 15). In COVID patients, the ACE2 protein level significantly increases in both alveolar tissue and bronchial epithelium of diabetic patients (16), and this can partly explain the high rate of infectivity of SARS-CoV-2 in some patients, as elderly and infants (17). In the SARS-CoV-2 pathophysiology, angiotensin-converting enzyme 2 (ACE2) seems to be the key cell receptor for SARS-CoV-2 infecting humans (18). SARS-CoV-2 uses its spike protein S1 to enter cells by interacting with the ACE2 receptor on the cell surface membrane. SARS-CoV-2 uses angiotensin-converting enzyme 2 (ACE2) as a major cell receptor to infect humans (19–22). SARS-CoV-2 infection interacts with ACE2 in lung tissue by binding with the spike (S) viral protein—a 1,273 amino acid-long protein (23). Another study has shown that the intestinal epithelium presents increased levels of ACE-2 protein and that the SARS-CoV-2 spike protein may have a major role by stimulating epithelial damages in the intestinal mucosa responsible for inflammation (24).

The link between ACE2 and the S-protein of SARS-CoV-2 results in the release of the RNA of SARS-CoV-2 into the host cell and in the convert of the viral genome RNA into replicase polyproteins 1ab and pp1a. Polyproteins 1ab and pp1a are cleaved into small products by proteinases (25). SARS-CoV-2Mpro plays a major role in the mechanism action of polyproteins (26).

Recently, Raj et al. reported, in their preliminary and *in vitro* study, that CBD can downregulate SARS-CoV-2 infection into two pathways (27) (**Table 1**). CBD can bind to SARS-CoV-2Mpro by blocking its transcription, and CBD can interact as an agonist of the CB2 receptor. These two activities can reduce the secretion of pro-inflammatory cytokines in lung cells (27).

**Table 1:** Actions of CBD use in different preclinical studies in COVID-19.

Property	Model	Findings	References
Anti-inflammatory	Human 3D skin artificial tissue model	Downregulation of COX2, TNF-α, IL-6, CCL2	(28)
Anti-inflammatory	Lung epithelial cell	Decrease cytokine secretion (IL-6, IL-8)	(29)
Anti-inflammatory	Intranasal Poly I :C-induced ARDS.	Decrease cytokine secretion	(30)
Anti-inflammatory	3D tissue models	Modulation of TMPRSS2 and ACE2 levels	(31)
Anti-inflammatory	Intranasal Poly I :C-induced ARDS.	Decrease cytokine secretion	(32)
Anti-inflammatory	Caco-2-cells	Decrease activity of SARS-CoV-2 spike protein by a PPARγ-dependent action	(33)
SARS-CoV-2 anti-replication	A549 human lung cells	Blockage in viral replication	(13)
SARS-CoV-2 anti-replication	Lung cells	Antagonism of SARS-CoV-2Mpro and agonism of CB2 receptor	(27)
Antiviral effect	Human lung fibroblasts	Preventive therapy	(34)

SARS-CoV-2, severe acute respiratory syndrome coronavirus-2; IL, interleukin; ACE2, angiotensin-converting enzyme 2; ARDS, acute respiratory disease syndrome; PPARγ, peroxisome proliferator-activated receptor gamma.

Moreover, CBD can activate the CB2 receptor to decrease the inflammatory macrophage release mechanism into the lungs (35) and can reduce the immune pathological mechanisms of viral infection (36). The SARS-CoV-2Mpro downregulation is not associated with side effects in humans and remains at this stage as one the best molecular targets for decreasing the coronavirus replication (37, 38). Thus, CBD, by acting as an agonist of the CB2 receptor, can decrease the activity of SARS-CoV-2Mpro and can downregulate the viral replication due to its binding affinity (27). At this date, SARS CoV-2Mpro inhibitors are not toxic in humans (26).

## CBD and the Cytokine Storm in SARS-CoV-2 Infection

The use of CBD can decrease the activity of inflammatory transcription factors, including AP-1, NF-kB, and NFAT pathways. This activity of CBD results in the decrease in the secretion of cytokines such as IL-6 and TNF-α (39). Moreover, the use of CBD in the murine model of asthma was associated with a decrease in different cytokines (40). Among COVID-19 patients, the use of CBD can be beneficial against the cytokine release syndrome but should be proved by clinical trials. CBD has been recently considered as a possible drug in the treatment of SARS-CoV-2 (41–43). This molecule can decrease the release of proinflammatory cytokines responsible for inflammation during SARS-CoV-2 infection (4). CBD can enhance the interferon pathway which leads to the activation of the host immune response to viral pathogens (13). The interferon pathway is a well-known signaling targeted as a possible treatment for COVID-19 (44). Moreover, recent findings highlighted the potential suppressor action of CBD on cytokine production in macrophages (45). Moreover, CBD can downregulate the expression of COX-2, TNF-α, IL-6, CCL2, and other cytokines in a WI-38 lung fibroblast cell line model with SARS-CoV-2 infection (28), as observed in a lung epithelial cell model with the decrease in IL-6 and IL-8 secretion (29) and in intranasal of Poly I:C-induced acute respiratory viral infection of COVID-19 (30) (**Table 1**).

## CBD and Antiviral Actions in SARS-CoV-2 Infection

CBD can decrease the activity of both TMPRSS2 enzymes and ACE2 acting in different viral gateways (i.e., oral, lung, intestinal epithelium) which are major issues for SARS-CoV-2 invasion (31). The possible action of CBD for the treatment of COVID-19 has been observed with the use of polycytidylic acid poly (I: C) (as a synthetic analogue of viral double-stranded RNA), inducing ARDS, in mice (32). Moreover, an *in vitro* investigation of a mixture of terpene with CBD use in human coronavirus E229i, as the combination of NT-VRL-1 (terpene-based formulation) with CBD, enhanced the antiviral effect (34). Furthermore, it was observed that CBD in combination with 7-OH-CBD can be used to lower SARS-CoV-2 infection occurring in patients (13) (**Table 1**).

## CBD and Perspectives in SARS-CoV-2 Infection

CBD appears to be a safe molecule for humans, as observed in different conducted trials (46). However, no data are published for the real efficacy and toxicity of CBD in humans for the COVID-19 disease. A lack of data exists to clearly state the use of CBD in SARS-CoV-2 infection as a therapy in addition to other drugs. Future clinical trials should be implemented to test the potential of CBD in COVID-19 patients to target the cytokine storm, the viral infection, and then the prevention of pulmonary fibrosis. In the different known pathological conditions, CBD efficacy depends on the dose and its bioavailability. Thus, different concentrations of CBD should be investigated to understand its possible effects. Moreover, the drug–drug interactions between CBD and other treatments are required to be investigated in COVID-19 patients, and at this stage a lack of studies remains still present. A recent study has shown that CBD could be a stronger antiviral agent than other drugs, such as lopinavir and remdesivir (27). Nevertheless, there are few research articles which highlight the potential action of CBD on cytokine storm in COVID-19. It is important to note that no unified therapy with CBD was still determined and the main part of the possible treatments remains experimental in other disorders (47). Moreover, it is important to demonstrate that CBD does not increase mortality among COVID-19 participants, as observed in infected pneumococcal meningitidis animals, with CBD administration, showing an increase in survival associated with a decrease in TNF-α expression (48).

## Two Pathways Could Explain the Possible Actions of CBD in SARS-CoV-2 Infection by Downregulating the Cytokine Storm

### CBD and PPARγ in SARS-CoV-2

A recent finding has shown that CBD can reduce both the expression of ACE-2 and RhoA-GTPase/Caspase-1/NLRP3 signaling through its interaction with PPARγ in Caco-2 cells, as intestinal epithelium cells *in vitro*. This interaction leads to counteract the viral entry and viral replication in SARS-CoV-2 infection (33). CBD exerts its activity through the interaction with PPARγ in COVID-19 infection (4, 33). PPARγ (peroxisome proliferator-activated receptor gamma) is a ligand-activated transcription factor which binds PPREs (PPAR-response elements). PPARγ is implicated in many pathophysiological mechanisms, including cell differentiation, protein metabolism, lipid metabolism, carcinogenesis (49), adipocyte differentiation, insulin sensitivity, and inflammation (50, 51). PPARγ ligands can be synthetic or natural, as CBD (4, 52, 53). PPARγ agonism in resident alveolar macrophages limits pulmonary inflammation and enhances host recovery following respiratory viral infections (54). PPARγ activation is responsible for the control of cytokine oversecretion with consequent amelioration of the tissue damages. COVID-19 survivors can develop postinfectious sequelae with persistently impaired lung function and pulmonary fibrosis (55). PPARγ receptors may be potential therapeutic targets in fibrotic lung diseases, due to their action of controlling fibroblast/myofibroblast activation and collagen secretion in murine models. Indeed, CBD can reduce pulmonary inflammation and fibrosis in animal models of asthma (40). CBD, as a PPARγ receptor agonist, could potentially be a therapeutic strategic way in COVID-19 patients. By a PPARγ-dependent signaling, CBD can prevent the inflammatory response of the SARS-CoV-2 spike protein in Caco-2-cells (33) (**Table 1**). This action is coupled with the inhibition of IL-1β, IL-6, IL-18, and TNF-alpha, responsible for the inflammatory process during SARS-CoV-2 infection (33).

## Hypothesis of CBD and WNT/β-Catenin Pathway in SARS-CoV-2

In parallel, the WNT/β-catenin pathway is upregulated in severe sepsis-induced acute lung injury and sepsis mouse models (56, 57). The WNT/β-catenin pathway is dysregulated in sepsis or ARDS and therefore plays a major role in fibrosis and inflammation (58, 59). In COVID-19 patients, the transforming growth factor (TGF-β) stimulates the WNT/β-catenin pathway, leading to an increased risk of pulmonary fibrosis (59) and pulmonary infection (60, 61). The name WNT is derived from Wingless drosophila melanogaster and its mouse homolog Int. The WNT/β-catenin pathway is involved in several mechanisms, controlling signaling, including embryogenesis, cell proliferation, migration and polarity, apoptosis, and organogenesis (62). The WNT/β-catenin pathway can be damaged in many pathological diseases, including inflammation, metabolic, neurological, and psychiatric disorders, fibrosis, and cancer (63, 64). Numerous findings have observed that the WNT/β-catenin pathway and PPARγ act in an opposing manner in disorders, such as chronic inflammation and fibrosis mechanisms (65, 66) and also SARS-CoV-2 infection (18). Numerous reports have shown that PPARγ agonists could be candidates for modulating the cytokine storm in the COVID-19 disease (67–69), whereas the WNT/β-catenin pathway can stimulate the cytokine storm release (for review, see (18)).

Moreover, the dysregulation in ACE2 expression in lung tissue may exacerbate outcomes in COVID-19 patients (70). In COVID-19 patients, ACE2 expression and the WNT/β-catenin pathway appeared to be interrelated (18). Rats with renal ischemia/reperfusion-induced injury tissue treated by pioglitazone, a PPARγ agonist, have shown a modulation of both ACE2 expression and WNT/β-catenin pathway decrease (71). Even though very few studies have so far shown the potential action of PPARγ agonists in the treatment of COVID-19, rosiglitazone can modulate ACE2 expression in animal models (72) and it may also potentially be utilized in diabetic patients with COVID-19 (73).

## Conclusion

The use of CBD to decrease the severity of the SARS-CoV-2 infection based on reported preclinical studies, in addition to the use of vaccines, should be investigated to reinforce the protection against COVID-19. We can hypothesize that CBD may have a possible action to counteract the inflammatory response in SARS-CoV-2 infection. Vaccines are the only way to prevent COVID-19, but it appears important to find therapeutic complements to treat patients already affected by SARS-CoV-2 infection. The possible effects of CBD through its relationship with both the WNT/β-catenin pathway and PPARγ expression should be investigated to better understand its downregulating role on the cytokine storm in SARS-CoV-2 infection. Moreover, more evidence is needed for the routine use of CBD in the treatment of COVID-19 and future clinical trials should be implemented to show its effectiveness.

## Data Availability Statement

The original contributions presented in the study are included in the article/supplementary material. Further inquiries can be directed to the corresponding author.

## Author Contributions

Conceptualization, AV. Writing—original draft preparation, AV. The author has read and agreed to the published version of the manuscript.

## Conflict of Interest

The author declares that the research was conducted in the absence of any commercial or financial relationship that could be construed as a potential conflict of interest.

## Publisher’s Note

All claims expressed in this article are solely those of the authors and do not necessarily represent those of their affiliated organizations, or those of the publisher, the editors and the reviewers. Any product that may be evaluated in this article, or claim that may be made by its manufacturer, is not guaranteed or endorsed by the publisher.
